# Impact of polyethylene fiber-reinforced composite resin and thermomechanical cycling on dentin bond strength

**DOI:** 10.1590/1807-3107bor-2025.vol39.013

**Published:** 2025-02-07

**Authors:** Maria Isabel Guimarães Carvalho Ribeiro PIMENTEL, Kamila Rosamilia KANTOVITZ, Cecília Pedroso TURSSI, Flávia Lucisano Botelho do AMARAL, Roberta Tarkany BASTING, Leandro de Moura MARTINS, Fabiana Mantovani Gomes FRANÇA

**Affiliations:** (a) Faculdade São Leopoldo Mandic, School of Dentistry, Department of Restorative Dentistry, Campinas, SP, Brazil.; (b) Universidade Federal do Amazonas – Ufam, Department of Restorative Dentistry, Manaus, AM, Brazil.

**Keywords:** Composite Resins, Polyethylene, Cycling, Dental Cements

## Abstract

This study evaluated the microtensile bond strength (μTBS) and fracture pattern of direct composite resin reinforced with polyethylene fiber (Ribbond^®^) on dentin substrate after thermomechanical cycling (TMC). Dentin blocks (dentin thickness=2 mm) were obtained from forty human third molars and randomly divided into four groups (n=10) according to type of restoration (composite resin with or without Ribbond^®^) and to whether they were or were not subjected to TMC (100,000 cycles of 50 N / 2 Hz / 1-minute baths of 5 and 55ºC). The 1-mm-thick square-shaped specimens were submitted to μTBS testing in a universal testing machine at 0.5 mm/min. The fracture patterns were assessed by stereoscopic magnifying glass (30X magnification). The μTBS (in MPa) and failure pattern data were subjected to the generalized linear model and G tests (a=0.05). Neither the polyethylene fiber nor TMC had any statistically significant effect (p=0.196 and p=0.136, respectively) on the μTBS of the composite resin to dentin. Adhesive failures were more prevalent in the composite resin group compared with the Ribbond-containing group when subjected to TMC. Additionally, the composite resin containing Ribbond^®^ showed a higher proportion of cohesive failures in composite resin than the resin groups not containing this fiber, irrespective of TMC. It was concluded that reinforcing the direct layer of composite resin with Ribbond^®^ polyethylene fiber did not influence the adhesive resistance to dentin, even when subjected to TMC. However, its incorporation did result in a higher frequency of cohesive failures in resin after TMC.

## Introduction

Composite resins are continually researched to enhance their ability to restore function and preserve remaining tooth structure.^1^ The good aesthetic characteristics and mechanical properties of these resins support their use in restorations for posterior teeth^1^. Some problems associated with composite restorations are insufficient toughness and polymerization shrinkage stress.^1^ Polymerization shrinkage is inevitable, since resin monomers bond to form a polymer network, becoming more pronounced with increasing cavity depth and larger volumes of restorative materials.^1,2^

Several methods can be used to compensate or reduce the effects of these polymerization shrinkage stresses in dental structures. One is an incremental technique for inserting composite resin into the cavity.^2^ Another is avoiding the creation of sharp angles during cavity preparation to minimize stress on both the tooth structure and the composite^2^. Another approach to further reduce polymerization shrinkage and provide the resin with greater support is to combine wedge-shaped resin increments with a fiber reinforcement system (Triaxial Ribbon).^3^ This combination also appears to help reduce the total volume of composite material needed.

The fiber-reinforced composite restoration was also introduced to increase durability, flexural strength,^4^ and fracture resistance of the composite restoration, and increase resin stiffness.^3,5,6^ It decreased polymerization shrinkage^7^ and provided better force distribution along the fibers,^3,5^ thereby improving the physical and mechanical properties of the composites when used with different types of dentin, including healthy dentin,^3-5^ affected dentin,^8^ and non-vital dentin.^9-11^ The strategic positioning of the fiber along the cavity walls can help prevent failure by distributing tension and absorbing energy. This configuration acts as a mechanism for blocking cracks and preserving the remaining tooth structure.^11,12^ In fact, the results of some studies have demonstrated that the incorporation of fibers into composite resins usually leads to more likely fracture patterns, especially above the cementoenamel junction, because the fiber layer acts as a voltage breaker and stops the propagation of cracks.^12-15^ Ribbond^®^ consists of high molecular weight polyethylene fibers with a thickness of 0.18 mm,^16^ and has been commercially available since 1992. It is made from pre-impregnated, silanized fibers treated with cold plasma.^6^ This plasma treatment increases the surface energy of the polyethylene fibers, enhancing their chemical activity.^4,17^ and ensuring better adhesion between the polyethylene and the resin.^6^

The selection of an appropriate treatment plan should be based on the remaining tooth structure, cavity wall thickness, position of the tooth in the arch, and the load applied to the tooth. Over the years, many techniques associated with these structural factors have emerged to improve fracture resistance and material adaptation, while reducing restoration stress.^5,9,15,18,19^ However, few studies have demonstrated the microtensile bond strength of direct fiber-reinforced composite resin on healthy dentin.^16^ Therefore, this in vitro study aimed to evaluate the microtensile bond strength and fracture pattern of direct composite resin incorporated with polyethylene fiber (Ribbond^®^) on a dentin substrate after thermomechanical cycling (TMC). The null hypotheses were: a) the presence of polyethylene fiber in the resin layer does not affect the microtensile bond strength to the dentin substrate; b) TMC does not affect the microtensile bond strength to the dentin; and c) the combination of polyethylene fiber (Ribbond^®^) and TMC does not affect the fracture pattern.

## Methods

### Experimental design

**Figure 1:**
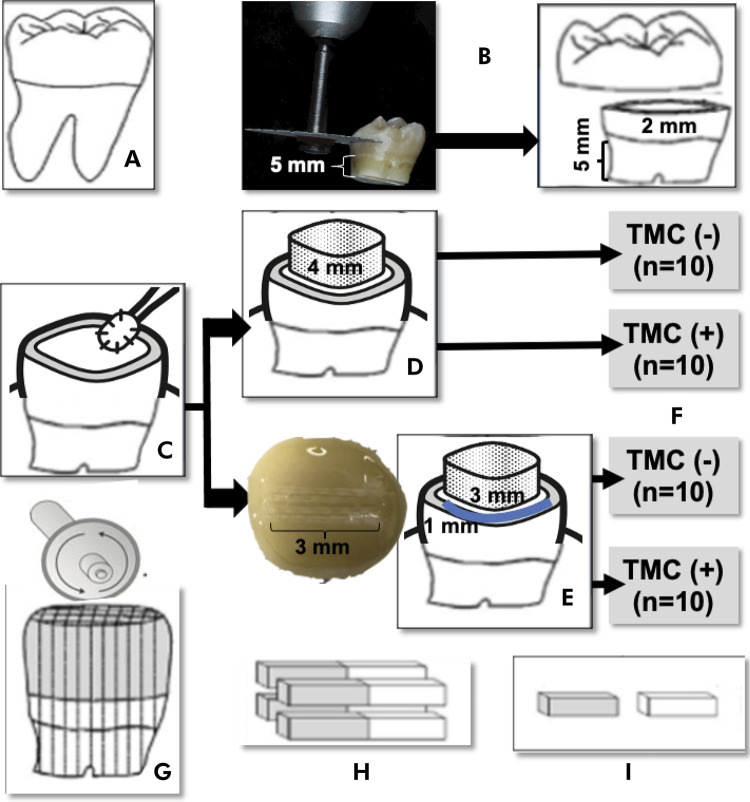
Representative scheme of methodology: (A) Tooth selection - 40 human third molars; (B) Preparation of tooth fragments (specimens); (C) Two-step self-etching; (D) Restorative procedure - resin coating technique with a 4-mm-high block; (E) Restorative procedure - resin coating technique containing fiber (RibbondÒ + flow composite resin = blue line ~ 1 mm in height); (F) Experimental groups with or without thermomechanical cycling (TMC = 100,000 mechanical cycles and 2,000 thermal cycles); (G) Longitudinal and vertical sectioning of the specimen into a series of 1-mm-thick squares; (H) Four central non-trimmed specimens; (I) Microtensile bond strength test (μTBS) and fracture pattern assessment.

This in vitro, prospective and qualitative-quantitative study was conducted after approval from the research ethics committee (CAEE. 59447422.6.0000.5374). The factors under study were two-level restoration with composite resin whether or not reinforced with polyethylene fiber (Ribbond^®^) subjected or not subject to thermomechanical cycling (TMC) (Figure 1). The experimental specimens consisted of 40 dentin blocks, randomly assigned to four treatment groups (n = 10), following the ADM guidelines for in vitro studies using teeth as the experimental unit.^12^ The response variables were microtensile bond strength (mTBS, in MPa) and type of fracture pattern (4 levels). This study followed the Academy of Dental Materials guidelines on in vitro testing of dental composite bonding to dentin.^20,21^

### Tooth collection and sample preparation

Extracted caries-free, non-restored human third molars were selected from individuals aged 16 to 40 years. These teeth were extracted for orthodontic reasons, and were free from apparent caries on the occlusal, buccal, palatal, and lingual surfaces.^20,21^ All the teeth were examined under 20X magnification (Leica MZ6; Wetzlar, Germany) to exclude those with any defect, erosive dentin, or sclerotic dentin (Figure 1A). The teeth were cleaned and stored in 0.5% chloramine T solution at 4ºC for up to 6 months after extraction.^20,21^ Their roots were sectioned 5 mm below the cementoenamel junction using a double-face diamond saw, and then discarded (KG Sorensen, São Paulo, Brazil). Subsequently, spherical steel drills #2 and #4 (Maillefer, SSWhite, Rio de Janeiro, Brazil) were used to remove the pulp chamber floor, followed by removal of the pulp tissue with curettes (Duflex, SSWhite, Rio de Janeiro, Brazil). Additionally, each tooth was sectioned vertically 2 mm above the cementoenamel junction (mid-dentin level) using the same method (Figure 1B). Dentin thickness was measured with a millimeter caliper (Golgran, São Caetano do Sul, Brazil) to ensure a uniform thickness of 2 mm during access to the pulp chamber. The teeth that did not fit into the range between 1.8 and 2.2 were replaced. The occlusal surface was polished with 600-grit silicon carbide (SiC) sandpaper and water under manual pressure for 15 s to form a dentin smear layer. After application of the adhesive system (Clearfil SE Bond, Kuraray, Tokyo, Japan), the remaining root was filled with composite resin (Z100, 3M/ESPE, Saint Paul, USA) to facilitate obtaining the specimens. The teeth were covered with wet tissue paper throughout the experimental process to maintain their hydration.^20,21^

The brand, manufacturer, and composition used in all the materials of the present study are described in Table 1. All the restorative procedures were completed in a temperature-controlled room (23 ± 2◦C). The two steps of the self-etching system (Clearfil SE Bond) were applied immediately after exposing the mild-dentin substrate and preparing the smear layer. First, the acid primer was actively applied to the dentin for 20 s using a microbrush (Figure 1C). Then, the surface was gently air-blown for 5 s with a triple syringe held 2 cm away to evaporate the solvent. A drop of adhesive was passively applied with a microbrush (20 s), and the excess was removed with a dry microbrush before being light-cured for 10 s. Regarding the composite resin group, Filtek Z350 XT was applied in 2 mm horizontal increments using a titanium resin spatula (Indusbello, Londrina, Brazil), and each increment was light-cured individually for 40 s to build up a block measuring 4 mm in height (Figure 1D). Regarding the composite resin containing Ribbond^®^, after the bonding procedure, 3 mm of the fiber was measured, cut with stainless steel Ribbond^®^ scissors, impregnated with one drop of the same adhesive, and applied in the middle of the flat surface of the specimen with a metal cotton plier. A 1-mm-thick layer of fluid resin was applied (GrandioSO heavy flow, VOCO, Cuxhaven, Germany), and the polyethylene fiber (Ribbond^®^, Seattle, USA) was positioned on the entire occlusal surface of the specimen before photoactivation, and then light-cured for 20 s. Subsequently, Filtek Z350 XT was applied in 2 mm horizontal increments to build a bonding surface block up to 4 mm in height (Figure 1E). Light-curing was carried out using the GranValo device (Ultradent Products, South Jordan, USA) in standard mode, 1000 mW/cm^2^, with the tip positioned up to 1 mm away from the composite resin restoration.

**Table 1:** Brand, manufacturer, and composition used for all the materials in the present study.

Manufacturing material	Brand	Local	Composition*
Clearfil SE Bond	Kuraray Noritake Dental	Tokyo, Japan	*Primer*: 10-MDP, HEMA, Hydrophilic dimethacrylate, N,N-diethanol p-toluidine, water
			*Adhesive*: 10-MDP, bis-GMA, HEMA, hydrophobic dimethacrylate, CQ, N,N-diethanol p-toluidine, silanated colloidal silica
Ribbond, Inc.	Oraltech	Seattle, WA, USA	Ultra-High Molecular Weight Polyethylene Fiber H-(CH_2_CH_2_) Homopolymer
GrandioSO heavy flow	VOCO GmbH	Cuxhaven, Germany	Shade A3 Barium aluminum borosilicate glass, silicon oxide, HEDMA, BisGMA, TEGDMA, BisEMA, fumed silicon oxide, initiators, stabilizers, coloring pigments
Filtek Z350 XT	3M ESPE	Saint Paul, MN, USA	Shade A3 Zirconia and silanized silica, UDMA, bisphenol A Polyethylene glycol diether methacrylate, Bis-GMA, TEGDMA, 2,6-di-tert-butyl-p-cresol

*BIS-GMA: bisphenol A glycidyl methacrylate; TEGDMA: triethylene glycol dimethacrylate; UDMA: Urethane dimethacrylate; 10 MDP: 10-methacryloyloxidecyl dihydrogen phosphate; HEMA: hydroxyethyl dimethacrylate; BIS-EMA: bisphenol hydroxyethyl methacrylate.

The mild-dentin specimens were randomly divided into four experimental groups according to the dental material, and subjected or not subjected to thermomechanical cycling (n = 10): Composite resin: restoration with nanoparticle resin (Z350, 3M ESPE, Saint Paul, USA); Ribbond^®^ + Composite resin: restoration with nanoparticle resin (Z350, 3M ESPE, Saint Paul, MN, USA) combined with fiber within the resin layer; Composite resin + TMC: restored with nanoparticle resin (Z350, 3M ESPE, Saint Paul, USA) subjected to TMC; Ribbond^®^ + Composite resin + TMC: restoration with nanoparticle resin (Z350, 3M ESPE, Saint Paul, USA) combined with fiber within the resin layer, and subjected to TMC (Figure 1F). The specimens were stored for 24 h at 37 ± 1^o^C and 50% relative humidity.

### Thermomechanical cycling (TMC)

The specimens were positioned inside the TMC machine (Elquip, São Carlos, Brazil) to receive a load in the axial direction on the restoration, and then subjected to 100,000 mechanical cycles with 50 N of load, 2 Hz frequency,^2^ and 2,000 thermal cycles of 5 and 55ºC lasting 30 s, corresponding to 6 months in the oral cavity.^21^ During the test, the specimens were kept at relative humidity and a temperature of 37^o^C.

### Preparation of specimens for the microtensile bond strength (μTBS) test

Afterward, each tooth was individually secured in acrylic resin plates (5 x 5 x 4 mm). Subsequently, the tooth was carefully aligned with the surface of interest, and sectioned longitudinally and vertically into a series of 1-mm-thick square specimens (also referred to as non-trimmed specimens^19^) by using a water-cooled diamond blade (Isomet, Buehler, Lake Bluff, USA). Four central non-trimmed specimens were obtained from each tooth (Figure 1).

### Microtensile bond strength (μTBS) test

The specimens were tested individually using an active gripping device (clamps). The μTBS test was performed in a universal testing machine (EZ test, Shimadzu, Kyoto, Japan) at a crosshead speed of 0.5 mm/min. The cross-sectional area at the fracture site was measured with a digital caliper (Mitutoyo, Suzano, Brazil), with an accuracy of 0.0001 mm. The load (in Kgf) and the bonding surface area of each specimen were recorded on a worksheet. The μTBS values were calculated in MPa, using the formula: R=F (kgf)/A (cm).^20,21^

### Fracture pattern assessment

The surfaces of each fractured specimen were examined using a stereoscopic magnifying glass (30X magnification) to classify them into four types of fracture patterns: 1 = adhesive failure; 2 = partial adhesive and partial cohesive failure (mixed); 3 = totally cohesive in resin; 4 =t otally cohesive in dentin. A blinded calibrated examiner (M.I.P) evaluated the failure pattern. The intraexaminer agreement level for the failure pattern was analyzed using Spearman’s correlation test, and was found to be 92%.

### Statistical analysis

The original data were analyzed using the Shapiro-Wilk normality and the Levene homoscedasticity tests. The μTBS data were submitted to the generalized linear model to investigate the effect of incorporating polyethylene fiber and performing TMC, while the failure pattern data were compared using the G test. The SPSS 23 (SPSS, Chicago, USA) and BioEstat 5.0 (Mamirauá Foundation, Belém, Brazil) software systems were used (a = 0.05).

## Results

Table 2 presents the mean values and standard deviations for the μTBS test. There was no statistically significant interaction between the polyethylene fiber (Ribbond^®^) and the TMC factors (p = 0.383). Similarly, no significant difference in μTBS was observed with either the polyethylene fiber (p = 0.196) or the TMC (p = 0.136).

**Table 2:** Means (standard deviation) of bond strength (MPa) of the composite resin to dentin with or without polyethylene fiber (Ribbond®) and thermomechanical cycling (TMC).

Experimental group	TMC		Overall average
	Without	With	
	MPa		
Composite resin	44.58 (9.68)	36.14 (13.60)	40.36 (12.28) A
Ribbond^®^ + Composite resin	36.87 (8.95)	34.62 (11.50)	35.75 (10.10) A
Overall average	40.72 (9.90) a	35.38 (12.29) a	–

Overall averages followed by the same uppercase letters indicate no significant difference among the groups whether with or without Ribbond^®^ (polyethylene fiber) (p = 0.196) using the generalized linear model. Overall averages followed by the same lowercase letters indicate no significant difference between thermocycling and non-thermocycling of specimens (p = 0.598) using the generalized linear model. There was no statistically significant interaction between incorporation or non-incorporation of polyethylene fiber and TMC (p = 0.383).

**Figure 2:**
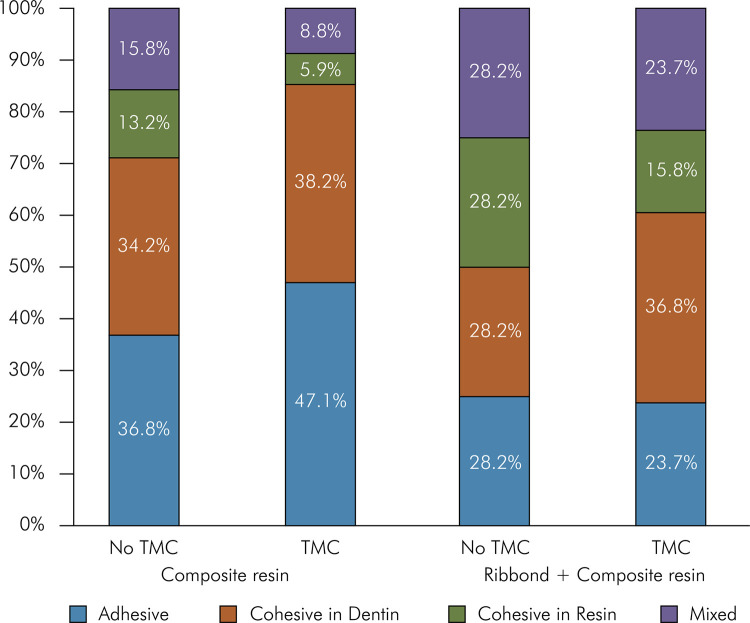
Relative frequency (%) of the failure pattern with and without polyethylene fiber and thermomechanical cycling (TMC)

The percentage (%) distribution of failure pattern detected in this study is shown in Figure 2. Adhesive failures are more prevalent in the composite resin group (47.1%), compared with the Ribbond-containing group (23.7%) when subjected to TMC. Furthermore, the composite resin group subjected to TMC exhibited the lowest percentage of cohesive failures in resin (5.9%). Additionally, the Ribbond-containing group showed a higher proportion of mixed and cohesive failures in resin than the composite resin groups without Ribbond, irrespective of TMC. The percentage of cohesive fracture in dentin was similar in all the experimental groups. The failure pattern did not differ significantly between the experimental and the non-TMC groups (p = 0.889).

## Discussion

The current investigation explored the effects of incorporating polyethylene fiber (Ribbon) into the resin layer on μTBS to the dentin substrate following TMC, as well as the fracture pattern. The first null hypothesis was not rejected. There was no difference in the μTBS to mild-dentin between fiber-reinforced and nanoparticle filler composites. The effectiveness of fiber reinforcement depends on many variables, such as the number of fibers within the resin matrix, and the type, length, form, and orientation (unidirectional, bidirectional, multidirectional) of the respective fibers.^23^ Other crucial aspects include adhesion to the polymer matrix, resin impregnation of the fiber device, and the adhesive system used.^24,25^ In fact, it is likely a culmination of these factors. A previous study demonstrated that bond strength to dentin is affected by the composition of the restorative material,^25^ attributed to either the mechanical properties^27^ or the surface-free energy characteristics of the composites.^25,28^ Another study emphasized that the dentin-bonding performance of the composites to dentin was influenced by the type of adhesive system.^29^ In the present study, the two-step self-etching procedure demonstrated effective chemical bonding of the 10-MDP functional monomer to calcium ions in hydroxyapatite, as corroborated by previous studies.^30^ This method seems to provide superior bonding efficacy, compared with that of the other adhesive systems.^31^ Furthermore, the present study aligns with a previous in vitro investigation that found no difference in microleakage and shear bond strength between Ribbon^®^ and twisted wire retainers bonded to human mandibular incisors using two different types of adhesives, both with and without primer,^32^ when particulate filler composite resin was used.^29^ Similarly, other studies have compared the fracture resistance of using polyethylene fiber in class II composite resin restorations, and report no significant difference between using and not using Ribbond^®^.^33,34^

Considering the clinical applications, this biomimetic approach may represent a less invasive and more conservative restorative option compared with other indirect restorative techniques using polymers. It is also a less costly and very promising technique to prevent fractures of extensive restorations in posterior teeth.^3,10,12^ The combination of polyethylene fiber with low-viscosity composite resin increases the ME of this dual approach from 13.97 MPa (only fiber) to 23.6 GPa (fiber combined with flowable resin).^20^ The high viscoelastic properties of this combination result in a plasticizing effect, which leads to a uniform distribution of stresses, and allows greater deformation before debonding.^7,15^ Ribbond^®^ is designed to transfer tension from the polymer matrix to the fibers, and thus act as a crack blocker,^15^ while the low viscosity of the flowable composite promotes effective diffusion into the pre-impregnated fiber network, and reduces the risk of bubble incorporation.^14^ Nonetheless, the present study found no difference in μTBS when Ribbond^®^ unidirectional fiber alignment was used with the flowable composite resin. One explanation for these results may relate to the technical sensitivity involved in the fiber reinforcement of the specimens.^34^ Polyethylene fibers must be impregnated with wetting or bonding adhesive before application. This adds a critical step to the clinical procedure. Voids within the matrix, or an excess of residual monomer can affect the interface between the fibers and the composite resin, potentially altering the properties of one or both materials, and leading to restoration failure.^7,35^ The findings of the present study corroborate those of other research^37^ that shows no significant difference in fracture resistance between fiber-reinforced and non-fiber-reinforced groups in endodontically treated teeth. However, using Ribbond^®^ as an occlusal splint increased the percentage of mixed and cohesive fracture patterns, thus better preserving the hybrid layer.

The microtensile test was performed in the current study to assess bond strength to mild-coronal dentin to explore its several advantages, such as its ability to obtain multiple specimens from a single tooth, and achieve more uniform stress distribution during loading across the interface, particularly for untrimmed specimens, such as those prepared for this study.^21^ It is important to note that the μTBS values measured in a low C-factor condition (flat dentin), ranging from 34.62 to 44.58 MPa, were significantly higher than the minimum bond strength values of 17 MPa, required for successful adhesion to dentin.^37^ Unlike the findings of Belli et al.,^38^ who demonstrated decreased dentin bond strength with flowable composite resin in high C-factor cavities, the combination of fiber with flowable resin can achieve stable bond strengths under a low C-factor condition. Specifically, polyethylene fiber combined with flowable resin can increase the μTBS to the dentin floor in high C-factor cavities.^38^

Simulation of the complex dynamics of oral conditions is crucial for applying in vitro studies to clinical practice, since it simulates the aging of restorations.^22^ This study hypothesized that TMC could increase tensions and weaken the adhesive bond of the teeth, potentially decreasing their bond resistance.^22^ However, this hypothesis was rejected. The present study found that TMC did not affect μTBS. This outcome could be attributed to the two-step self-conditioning adhesive (Clearfil SE Bond) used in the present study, which maintained a high μTBS^39^ even after the restorations were subjected to TMC. Despite the presence of fiber in the adhesive layer within the low-viscosity resin, μTBS depends primarily on the hybrid layer created by the adhesive system. The presence of this hybrid layer is crucial for maintaining adhesion stability.^30^ It should be noted that the present in vitro study was based on a simple mechanical movement that does not accurately mimic the three-dimensional natural chewing movements involving different forces and directions. Nonetheless, it affords insight into the basic understanding of the μTBS of the technique tested. Furthermore, the study results can serve as a reference for future studies employing a more dynamic environment.

The combination of Ribbond^®^ and TMC did not affect the fracture pattern; therefore, the third null hypothesis failed to be rejected. Nevertheless, it was found that the percentage of adhesive failure was predominant when the fiber was not included in the flowable composite resin. In contrast, the presence of fiber reinforcement resulted in predominantly mixed or cohesive failure patterns. These types of failures may indicate that the hybrid layer was preserved in the presence of the fiber. The resistance of the resin to rapid crack propagation, known as toughness, could be enhanced in composite resin by reinforcing the fiber, and thus increasing its durability and rigidity, while providing better force distribution along the fibers.^9^ Conversely, the inclusion of the fiber might weaken the resin material by introducing internal stresses, and lead to cohesive fracture patterns. Furthermore, the adhesive system may have interfered with the mechanical properties by accumulating monomers in the fiber, and increasing the proportion of organic matrix. This could have created a more fragile interface. It is important to highlight that no similar studies were found on μTBS to dentin using polyethylene fiber and TMC, thus indicating that further research is needed to investigate the influence of polyethylene fiber on fracture patterns.

## Conclusions

Incorporation of Ribbond^®^ polyethylene fiber into the resin layer did not affect the microtensile strength to dentin, even when the resin was subjected to thermomechanical cycling. However, its incorporation did result in a higher frequency of cohesive fractures in resin after thermomechanical cycling.
